# Interfascial Plane Blocks for Perioperative Analgesia in Dogs Undergoing Laparoscopic Ovariectomy: A Retrospective Study (2020–2025)

**DOI:** 10.3390/ani16111612

**Published:** 2026-05-26

**Authors:** Andrea Paolini, Matteo Serpieri, Giuseppe Bonaffini, Roberto Tamburro, Amanda Bianchi, Giuseppe Annunziata, Mitzy Mauthe von Degerfeld

**Affiliations:** 1 Department of Veterinary Sciences, University of Teramo, Strada Provinciale 18, 64100 Piano d’Accio, TE, Italy; apaolini@unite.it (A.P.); rtamburro@unite.it (R.T.); abianchi@unite.it (A.B.); giuseppe.annunziatavet@gmail.com (G.A.); 2 Department of Veterinary Sciences, University of Turin, Largo Braccini 2, 10095 Grugliasco, TO, Italy; giuseppe.bonaffini@unito.it (G.B.); mitzy.mauthe@unito.it (M.M.v.D.); 3 Vetplus—Clinica Veterinaria e Pronto Soccorso, Sant’Antimo, Via Degli Oleandri 50, 80029 Sant’Antimo, NA, Italy

**Keywords:** locoregional anaesthesia, perioperative analgesia, caudal thoracic paravertebral block, quadratus lumborum block, transversus abdominis plane block, dog

## Abstract

This retrospective study compared three ultrasound-guided locoregional techniques—transversus abdominis plane (TAP), quadratus lumborum (QL), and caudal thoracic paravertebral (C-TPV) blocks—in dogs undergoing laparoscopic ovariectomy. Ninety-eight dogs were included. The primary outcome was intraoperative rescue opioid requirement, while secondary outcomes included block execution time, postoperative analgesic requirements, intraoperative hypotension, and block-related complications. QL showed the shortest block execution time, followed by C-TPV and TAP. Intraoperative fentanyl requirements differed significantly among groups, with the highest requirements observed in the TAP group, intermediate values in the QL group, and the lowest in the C-TPV group. Postoperative methadone requirements were minimal across groups, with a statistically significant difference observed only between C-TPV and TAP. Rates of intraoperative hypotension and block-related complications were low and comparable among techniques. In this retrospective cohort, C-TPV was associated with lower intraoperative opioid requirements, whereas QL showed the shortest execution time. Postoperative differences were minimal and of uncertain clinical relevance. Prospective controlled studies are warranted to confirm these findings.

## Simple Summary

Ultrasound-guided locoregional techniques are increasingly used to improve perioperative analgesia in dogs undergoing laparoscopic procedures. Among these, transversus abdominis plane (TAP), quadratus lumborum (QL), and caudal thoracic paravertebral (C-TPV) blocks are commonly applied, although their comparative performance remains unclear. This retrospective study evaluated 98 dogs undergoing laparoscopic ovariectomy to compare these three techniques in terms of execution time, analgesic requirements, and perioperative safety. QL was associated with the shortest execution time, whereas C-TPV was associated with lower intraoperative opioid requirements. Postoperative analgesic requirements were minimal across groups, with only limited differences observed. Rates of hypotension and block-related complications were low and comparable among techniques. These findings suggest that all three techniques may provide clinically relevant perioperative analgesia, with differences mainly observed in intraoperative opioid requirements and procedural characteristics. However, prospective controlled studies are needed to confirm these results.

## 1. Introduction

Laparoscopic ovariectomy is increasingly performed in canine clinical practice because it is associated with reduced surgical trauma, faster recovery, and improved perioperative comfort compared with traditional open approaches [1,2,3]. However, laparoscopic surgery does not eliminate nociceptive input. Pain and sympathetic stimulation may arise from trocar placement, abdominal wall distension, pneumoperitoneum, and ovarian manipulation [4,5]. Therefore, adequate perioperative analgesia remains an essential component of anaesthetic management in dogs undergoing laparoscopic ovariectomy.

Ultrasound-guided locoregional anaesthesia has been increasingly incorporated into multimodal analgesic protocols in small animal practice. Interfascial plane blocks aim to deposit local anaesthetic within defined anatomical planes, allowing spread toward segmental nerves involved in the abdominal wall and, depending on the technique, potentially visceral nociceptive transmission.

The *transversus abdominis* plane (TAP) block involves injection of local anaesthetic between the internal abdominal oblique and *transversus abdominis* muscles, targeting the ventral branches of the thoracolumbar nerves supplying the abdominal wall and parietal peritoneum. In dogs, cadaveric studies demonstrated that the extent of nerve staining and injectate spread may vary according to injection site and technique, leading to the development of lateral, subcostal, and combined approaches to improve abdominal wall coverage [6,7,8,9,10]. Clinical studies suggest that TAP block may reduce perioperative nociception and postoperative analgesic requirements in abdominal procedures, including laparoscopic ovariectomy [11,12,13]. However, the TAP block is generally considered to provide predominantly somatic abdominal wall analgesia, whereas modulation of visceral nociception may be less predictable.

The *quadratus lumborum* (QL) block deposits local anaesthetic adjacent to the *quadratus lumborum* muscle, with possible spread toward thoracolumbar ventral branches and adjacent sympathetic structures. Previous cadaveric and clinical studies in dogs indicate that this block is feasible and may provide perioperative analgesia for abdominal surgery [14,15,16].

The caudal thoracic paravertebral (C-TPV) block involves local anaesthetic injection within the thoracic paravertebral space, close to the emerging spinal nerves. In dogs, thoracic paravertebral approaches have been described in cadaveric and clinical studies and may provide segmental analgesia relevant to thoracic and cranial abdominal procedures [17,18].

In clinical practice, the choice among these techniques is often influenced by the expected extent of analgesic coverage, technical complexity, operator familiarity, and the perceived balance between somatic and visceral analgesia. The TAP block is commonly selected because of its relative technical simplicity, reproducibility, and established role in abdominal wall analgesia. In contrast, QL and C-TPV blocks are generally considered more technically demanding but may provide a more proximal spread of local anaesthetic and potentially greater modulation of visceral nociceptive pathways. Consequently, clinicians may preferentially select different techniques depending on the type of abdominal procedure, anticipated nociceptive component, and individual operator experience. However, comparative clinical data supporting these decision-making processes in dogs undergoing laparoscopic ovariectomy remain limited [19,20].

Despite the increasing clinical use of these techniques, comparative data in dogs undergoing laparoscopic ovariectomy remain limited. Most available evidence is derived from cadaveric, experimental, or small prospective studies, and less information is available on real-world clinical application, including execution time, analgesic requirements, and perioperative complications.

Therefore, this retrospective study aimed to compare TAP, QL, and C-TPV blocks in dogs undergoing laparoscopic ovariectomy, focusing on block execution time, intraoperative and postoperative rescue analgesia, hypotension, and block-related complications.

## 2. Materials and Methods

### 2.1. Study Design and Case Selection

This retrospective observational study evaluated the clinical application of three ultrasound-guided locoregional techniques in dogs undergoing elective laparoscopic ovariectomy. Medical records of client-owned dogs referred for laparoscopic ovariectomy over a five-year period (July 2020 to December 2025) were reviewed. Dogs were included if laparoscopic ovariectomy was performed in combination with one of the following locoregional techniques: transversus abdominis plane (TAP), quadratus lumborum (QL), or caudal thoracic paravertebral (C-TPV) block. Cases converted to open surgery or with incomplete clinical records were excluded. Cases were collected from the Veterinary Teaching Hospital of the University of Teramo and a private clinical practice.

All anaesthetic procedures were performed by two veterinarians (A.P. and G.A.) with advanced training and routine clinical experience in ultrasound-guided locoregional anaesthesia. The choice of block technique was based on operator preference and clinical practice at the time of the procedure, reflecting real-world conditions rather than a standardized allocation protocol. Only dogs classified as American Society of Anesthesiologists (ASA) physical status I, based on physical examination and routine preoperative laboratory testing, were included. Additional inclusion criteria were regular vaccination status, absence of pregnancy, and absence of oestrus at the time of surgery. All procedures were performed as part of standard clinical practice. Written informed consent was obtained from all owners prior to anaesthesia and surgery.

Ethical approval by an institutional review board was not required, as no experimental procedures were performed, and all anaesthetic techniques were routinely used in dogs.

### 2.2. Transversus Abdominis Plane (TAP) Block

According to the technique described by Schroeder et al. [8], the TAP block was performed with the animals in dorsal recumbency following aseptic skin preparation. The ultrasound transducer was placed transversely on the lateral abdominal wall to identify the external abdominal oblique, internal abdominal oblique, and transversus abdominis muscles. The needle was advanced until the fascial plane between the internal oblique and transversus abdominis muscles was reached.

Correct positioning was confirmed by negative aspiration and real-time ultrasonographic visualization of local anaesthetic spread within the target plane. The procedure was repeated on the contralateral side to achieve bilateral blockade.

### 2.3. Quadratus Lumborum (QL) Block

The QL block was performed with the dog in lateral recumbency following clipping of the thoracolumbar region as described by Garbin et al. [14]. The ultrasound transducer was placed caudal and parallel to the last rib to obtain a transverse view of the lumbar musculature. Key ultrasonographic landmarks included the transverse process of the first lumbar vertebra, the quadratus lumborum muscle, and the psoas muscle. Using an in-plane approach, the needle was advanced until the tip was positioned within the interfascial plane between the quadratus lumborum and psoas muscles.

Correct placement was confirmed by real-time ultrasonographic visualization of injectate spread within the target plane. The procedure was repeated on the contralateral side.

### 2.4. Caudal Thoracic Paravertebral (C-TPV) Block

The C-TPV block was performed according to the technique described by Ferreira et al. [21]. Dogs were positioned in sternal recumbency, and a sagittal in-plane ultrasound approach was used. The transducer was placed lateral to the spinous processes at the caudal thoracic level to visualize the transverse processes and pleura. The needle was advanced in a craniocaudal direction until positioned within the thoracic paravertebral space, immediately dorsal to the pleura and adjacent to the spinal nerve.

Correct placement was confirmed by real-time visualization of ventral pleural displacement during injection.

### 2.5. Anaesthetic and Perioperative Management

All dogs were managed according to a standardized anaesthetic and perioperative protocol applied in both clinical settings. Pre-anaesthetic evaluation included physical examination and routine laboratory testing (complete blood count and serum biochemistry). Food was withheld for 8 h prior to anaesthesia, while water was available until premedication.

Premedication consisted of methadone (Semfortan 10 mg mL^−1^, Eurovet Animal Health BV, Bladel, Netherlands; 0.2–0.3 mg kg^−1^ IM) and dexmedetomidine (Dexdomitor 0.5 mg mL^−1^, Orion Corporation, Espoo, Finland; 3–5 µg kg^−1^ IM). After sedation, an intravenous catheter was placed in a cephalic vein under aseptic conditions. Pre-oxygenation was provided for 3 min via flow-by (100 mL kg^−1^ min^−1^). General anaesthesia was induced with intravenous propofol (Proposure 10 mg mL^−1^, Boehringer Ingelheim Animal Health, Lyon, France) titrated to effect and maintained with isoflurane (Isoflo, Zoetis Italia S.r.l., Milan, Italy) in a 50:50 oxygen–air mixture. Intraoperative monitoring included heart rate (ECG), respiratory rate (capnography), non-invasive arterial blood pressure (SAP, MAP, DAP), oesophageal temperature, peripheral oxygen saturation (SpO_2_), end-tidal carbon dioxide (EtCO_2_), and end-tidal isoflurane concentration (EtISO). All procedures were initiated at a standardized EtISO of 1.1%. Lactated Ringer’s solution (Ringer Lattato, B.Braun Milano S.p.a., Milan, Italy) was administered intraoperatively at 3 mL kg^−1^ h^−1^.

Ultrasound-guided blocks were performed using a linear 10-MHz probe (Chison Viatom, Chison, Wuxi, China) and 22G echogenic insulated needles (Stimuplex^®^ Onvision, B. Braun, Milan, Italy). Ropivacaine 0.5% (Ropivacaina Galenica Senese, Industria Farmaceutica Galenica Senese S.r.l., Monteroni d’Arbia, SI, Italy) was administered at a total dose of 4 mg kg^−1^. When bilateral blocks were performed, a total volume of 0.8 mL kg^−1^ was divided equally between sides. In dogs receiving bilateral QL or C-TPV blocks, the total volume was distributed equally between sides (0.4 mL kg^−1^ per side). In the TAP subgroup receiving the four-point technique, 0.2 mL kg^−1^ was injected at each site, maintaining a total volume of 0.4 mL kg^−1^ per hemiabdomen. In the TAP group, most dogs received a single injection per side, while a subset (*n* = 17) received a four-point technique. Block execution time was defined as the interval from completion of aseptic preparation to complete withdrawal of the needle.

A block was considered successfully performed when the entire calculated volume of local anaesthetic was delivered within the intended target plane under ultrasound visualization. Complications were defined as failure to correctly deposit the injectate within the target plane or inadvertent puncture of adjacent structures. A 5 min interval was allowed between block execution and surgical stimulation. Laparoscopic ovariectomy was performed using a standardized three-port technique [22].

Intraoperative nociception was defined as an increase of ≥20% in at least two of the following parameters compared with the preceding 5 min interval: heart rate, respiratory rate, or mean arterial pressure. In such cases, rescue analgesia was administered with fentanyl (Fentadon, 50 µg mL^−1^, Eurovet Animal Health BV, Bladel, Netherlands; 1 µg kg^−1^ IV). Hypotension was defined as a mean arterial pressure (MAP) < 60 mmHg and was managed according to clinical judgment after assessment of anaesthetic depth. Physiological variables, rescue analgesic administrations, block-related events, and block execution times were recorded in the anaesthetic record and retrospectively extracted for analysis.

Postoperative pain was assessed every 2 h using the Glasgow Composite Measure Pain Scale—Short Form Italian validated version (CMPS-ISF) [23], starting from recovery to sternal recumbency and continuing throughout hospitalization. Dogs were routinely monitored for approximately 6 h postoperatively before same-day discharge, according to the standard clinical protocol of the participating centres. Rescue analgesia consisted of methadone (0.2 mg kg^−1^ IV) administered when CMPS-ISF scores reached ≥6/24 (or ≥5/20 without the mobility item). The evaluator was not blinded to the block technique. At discharge, all dogs received meloxicam (Meloxidyl, 5 mg mL^−1^, Ceva Salute Animale S.p.A., Milan, Italy; 0.2 mg kg^−1^ SC) as a single dose.

### 2.6. Statistical Analysis

Descriptive statistics and analyses were conducted using R (RStudio, version 2025.05.1+513). Continuous non-normally distributed variables are presented as median and interquartile range (IQR), whereas categorical data are expressed as counts and percentages.

Between-group homogeneity for age, body weight, and block execution time was evaluated using the Kruskal–Wallis test, followed by Benjamini–Hochberg-adjusted post hoc pairwise comparisons when appropriate. Breed distribution was summarized descriptively only, as the high number of categories and sparse counts across groups did not support a reliable inferential comparison.

Categorical outcomes, including block-related complications, hypotension, and the proportion of dogs requiring at least one rescue analgesic administration, were compared among groups using Fisher’s exact test (two-tailed), with BH-adjusted pairwise comparisons when appropriate. Continuous outcomes, including premedication drug dosages and block execution time, were compared using the Kruskal–Wallis test followed by BH-adjusted pairwise comparisons. Statistical significance was defined as *p* < 0.05.

## 3. Results

### 3.1. Study Population

**Figure 1:**
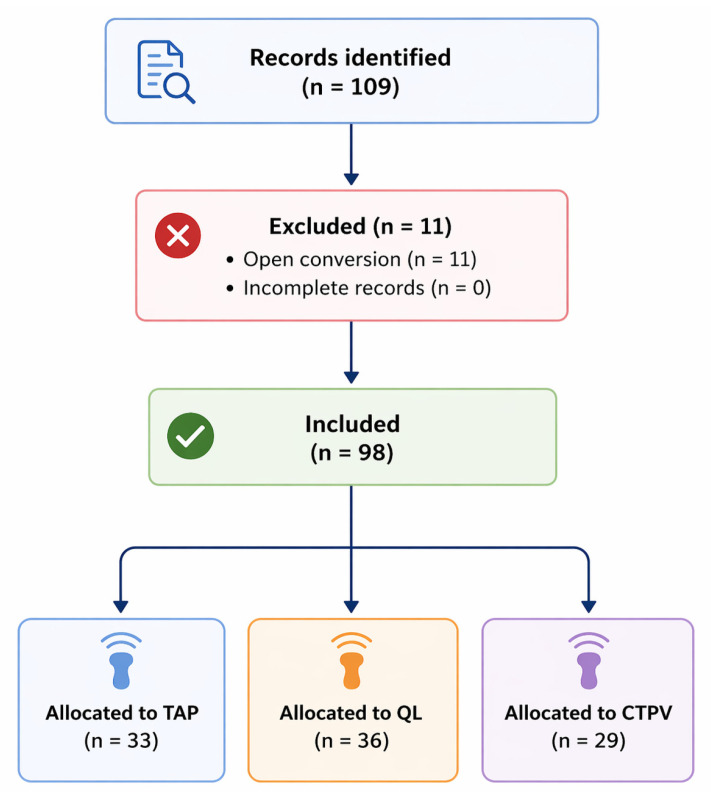
Study flow diagram of case selection and allocation. A total of 109 records were initially identified. Eleven cases were excluded due to open surgical conversion (*n* = 11), while no cases were excluded for incomplete records. Consequently, 98 cases met the inclusion criteria and were included in the analysis. These were grouped accordingly into three groups based on the locoregional anaesthesia technique performed: transversus abdominis plane block (TAP, *n* = 33), quadratus lumborum block (QL, *n* = 36), and caudal thoracic paravertebral block (C-TPV, *n* = 29).

A total of 109 dogs undergoing laparoscopic ovariectomy during the study period were initially identified (Figure 1). Of these, 11 cases were excluded due to conversion to open surgery, resulting in 98 dogs included in the final analysis (TAP *n* = 33, QL *n* = 36, C-TPV *n* = 29).

Age differed significantly among groups (Kruskal–Wallis, *p* = 0.01). Post hoc analysis showed a significant difference between the QL and C-TPV groups (BH-adjusted *p* = 0.02), whereas no significant differences were detected between TAP and QL or between TAP and C-TPV (Table 1).

Body weight did not differ significantly among groups (Kruskal–Wallis, *p* = 0.91) (Table 1). Breed distribution was heterogeneous across groups and was therefore reported descriptively only. Overall, mixed-breed dogs were the most represented breed category (25/98, 25.5%), followed by Abruzzese Shepherds (10/98, 10.2%), French Bulldogs (9/98, 9.2%), German Shepherds (8/98, 8.2%), Yorkshire Terriers (5/98, 5.1%), Cockers (4/98, 4.1%), and Cane Corsos (4/98, 4.1%). In the TAP group (*n* = 33), mixed-breed dogs were the most frequent (9/33, 27.3%), followed by German Shepherds (4/33, 12.1%) and French Bulldogs (4/33, 12.1%). In the QL group (*n* = 36), Abruzzese Shepherds (6/36, 16.7%) and mixed-breed dogs (6/36, 16.7%) were the most represented, followed by Yorkshire Terriers (4/36, 11.1%). In the C-TPV group (*n* = 29), mixed-breed dogs predominated (10/29, 34.5%), followed by Abruzzese Shepherds (4/29, 13.8%); German Shepherds, Bull Terriers, Lagotti, and French Bulldogs each accounted for 2/29 dogs (6.9%).

### 3.2. Premedication and Block Execution Time

All dogs received premedication with methadone and dexmedetomidine. Methadone dosage did not differ significantly among groups, whereas dexmedetomidine dosage showed a statistically significant difference between groups. Block execution time differed significantly among groups (Kruskal–Wallis test, *p* < 0.01). The QL block required a significantly shorter execution time compared with both TAP and C-TPV blocks. The TAP block was associated with the longest execution times, while C-TPV block execution times were intermediate between TAP and QL, as reported in Table 1.

### 3.3. Hemodynamic Variables and Adverse Events

The incidence of complications did not differ significantly among the three groups. Two events occurred in the TAP group and consisted of inadvertent intraperitoneal needle advancement beyond the transversus abdominis muscle. Both were immediately recognized under ultrasound guidance and corrected without clinical consequences. A single event was documented in the C-TPV group, consisting of partial deposition of local anaesthetic superficial to the endothoracic fascia, requiring needle repositioning to achieve appropriate spread within the paravertebral space. No complications were observed in the QL group. Similarly, no significant differences were observed in the occurrence of hypotension either within 20 min after block performance or during the intraoperative period.

Block-related complications were infrequent and comparable across groups, with no statistically significant differences detected. Data regarding the adverse events are reported in Table 2.

### 3.4. Perioperative Analgesic Requirements

The proportion of dogs requiring at least one intraoperative fentanyl rescue differed significantly among groups (Fisher’s exact test, *p* < 0.01). Rescue fentanyl was required most frequently in the TAP group, less frequently in the QL group, and was not required in any dog receiving C-TPV. Pairwise comparisons were significant between all groups (Table 3).

The proportion of dogs requiring at least one postoperative methadone rescue also differed among groups (Fisher’s exact test, *p* = 0.02). A significant pairwise difference was observed only between TAP and C-TPV, whereas no significant differences were detected between TAP and QL or between QL and C-TPV (Table 3).

## 4. Discussion

Based on the clinical cases collected over the five-year study period, TAP, QL, and C-TPV appeared to be feasible and clinically applicable locoregional anaesthetic techniques for perioperative analgesic management in dogs undergoing laparoscopic ovariectomy. In the present cohort, block-related complications were recorded in 3/98 dogs (3.1%), and all events were minor and immediately corrected without clinical sequelae. This incidence appears low and is consistent with the generally favourable safety profile reported for ultrasound-guided abdominal wall, quadratus lumborum, and paravertebral techniques in dogs, although direct comparisons are limited by differences in study design, definitions of complications, operator experience, and clinical setting [8,9,24,25,26,27,28,29].

Among the techniques, C-TPV was associated with lower intraoperative opioid requirements compared with TAP and QL, whereas postoperative differences were limited. This finding may reflect differences in anatomical spread and potential visceral coverage, although such mechanisms cannot be directly confirmed in the present study. Accordingly, the following discussion distinguishes findings directly observed in the present cohort from anatomical and mechanistic interpretations derived from previous literature.

### 4.1. Premedication and Block Execution Time

The retrospective design of the study resulted in minor variability in premedication dosing. Methadone administration did not differ significantly among groups and remained within a narrow clinical range (0.2–0.3 mg kg^−1^ IM), making a major confounding effect on perioperative nociception unlikely [30,31,32].

Dexmedetomidine dosage, however, showed a statistically significant overall difference among groups, although the absolute dose range was small (3–5 µg kg^−1^ IM) and only the C-TPV versus TAP comparison reached pairwise significance. Because dexmedetomidine exerts antinociceptive and anaesthetic-sparing effects [33,34,35], a residual confounding influence on intraoperative fentanyl requirements cannot be excluded. However, the group with the lowest fentanyl requirements did not receive the highest dexmedetomidine dosage, and all procedures were initiated at a standardized end-tidal isoflurane concentration (EtISO 1.1%). Accordingly, dexmedetomidine may have contributed to nociceptive modulation, but it is unlikely to fully account for the observed between-group differences.

Block execution time also differed among techniques, with QL showing the shortest median duration, C-TPV intermediate values, and TAP the longest times. These differences may reflect the sonographic window and technical complexity of each approach, as well as the partial heterogeneity of the TAP group, in which a subset of dogs received a four-point technique. Therefore, execution time findings should be interpreted as pragmatic indicators of procedural efficiency rather than definitive measures of technical superiority.

### 4.2. Complications and Hemodynamic Variables

Rates of early post-block and intraoperative hypotension were low and did not differ significantly among the groups. Block-related complications were rare, with only three events recorded in the entire cohort, all of which were minor and immediately corrected. In our interpretation, these events were likely related to the technical characteristics of the respective approaches rather than to clinically relevant adverse effects. TAP, QL, and C-TPV blocks require accurate ultrasonographic identification of fascial planes and adjacent anatomical structures, and small variations in needle trajectory or tissue characteristics may influence injectate deposition. This interpretation is supported by previous anatomical and clinical descriptions of these techniques, which emphasize the importance of ultrasound-guided needle placement and real-time visualization of injectate spread [8,9,24,25,26,27,28,29].

The low incidence of complications observed in the present cohort is consistent with the generally favourable safety profile reported for ultrasound-guided interfascial and paravertebral techniques in dogs [8,9,24,25,26,27,28,29]. From a mechanistic perspective, these approaches may be less likely than neuraxial techniques to produce clinically relevant sympathetic blockade, which could partly explain the limited haemodynamic effects observed in this study [36,37,38,39]. However, because of the retrospective design and the limited number of adverse events, these findings should be interpreted cautiously.

### 4.3. Perioperative Analgesic Requirements

In the present cohort, all three blocks were associated with low postoperative opioid requirements. However, intraoperative rescue analgesia differed among the groups. The proportion of dogs requiring at least one intraoperative fentanyl rescue was highest in the TAP group, intermediate in the QL group, and lowest in the C-TPV group. These represent direct observations from the present study.

A possible explanation for this pattern may be derived from the anatomical characteristics of the respective techniques. Based on previous anatomical and clinical studies, the TAP block is considered to provide predominantly somatic abdominal wall analgesia and may have limited ability to modulate visceral afferent pathways [15,16,25,29]. In contrast, QL and paravertebral approaches may allow local anaesthetic spread toward spinal nerve branches and adjacent structures potentially involved in visceral nociceptive modulation [14,17,18]. This interpretation is clinically plausible because laparoscopic ovariectomy involves visceral nociceptive input related to pneumoperitoneum and ovarian manipulation [2,4]. Therefore, the lower intraoperative rescue requirement observed in the C-TPV group may be related to a more proximal segmental effect. However, anatomical spread was not assessed in the present study, and this explanation remains speculative.

Another possible contributor is the systemic absorption of local anaesthetics. Previous studies indicate that interfascial techniques using relatively large injectate volumes may result in measurable systemic local anaesthetic concentrations [25,28,40,41,42,43]. Therefore, the analgesic profile observed in the present cohort may reflect a combination of local neural blockade and systemic pharmacological effects. However, plasma ropivacaine concentrations were not measured, and the relative contribution of systemic absorption cannot be determined from these data.

In the postoperative phase, the proportion of dogs requiring methadone rescue was low across all groups. Although a statistical difference was detected between TAP and C-TPV, the absolute frequency of rescue administration was low, suggesting limited clinical relevance. We interpret this postoperative convergence among groups as possibly related to the reduction in visceral nociceptive input after resolution of pneumoperitoneum and ovarian manipulation. In this phase, residual pain may be more strongly influenced by abdominal wall and trocar-site somatic nociception, for which all three techniques may provide some degree of coverage [2,4,44,45]. However, this mechanism was not directly assessed in the present study and should therefore be considered speculative.

Overall, the main finding directly observed in this cohort was the difference in intraoperative rescue opioid administration among techniques, whereas postoperative rescue requirements were low and broadly similar. The anatomical explanations proposed above should be considered interpretative and require confirmation in prospective studies designed to assess block spread and analgesic mechanisms.

### 4.4. Limitations

This study has several limitations inherent to its retrospective design. First, the absence of randomization and the lack of a standardized allocation protocol for block selection may have introduced selection bias.

The choice of locoregional technique may have been influenced by operator preference, clinical context, or temporal factors, potentially affecting group comparability. Second, cases were collected from two clinical settings and managed by different anaesthetists over a five-year period. Although this reflects real-world clinical practice, it may have introduced variability related to operator experience, centre-specific protocols, and potential learning curve effects, which may have influenced block execution time and procedural performance. Third, minor differences in premedication protocols, particularly in dexmedetomidine dosing, could have influenced nociceptive responses. These differences were within a narrow clinical range; however, a residual confounding effect cannot be excluded. Fourth, the TAP group was not entirely homogeneous, as a subset of dogs received a four-point injection technique. This variability may have influenced both execution time and analgesic performance. Fifth, postoperative pain assessment was based on clinical scoring systems and rescue analgesia requirements, which, although widely used, may not fully capture subtle differences in pain perception among groups.

In addition, postoperative pain assessment was not performed by a blinded evaluator, which may have introduced some degree of observer bias in pain scoring and rescue analgesia decisions, particularly when comparing postoperative analgesic requirements among groups. Moreover, a fixed 5 min interval was allowed between block performance and surgical stimulation for all techniques. Because onset time may differ among locoregional approaches, this standardized interval may have favoured or penalized specific techniques and could have influenced intraoperative rescue opioid requirements.

Finally, the study did not include a control group without locoregional anaesthesia, limiting the ability to draw definitive conclusions regarding the absolute efficacy of each technique. For these reasons, the results should be interpreted as associative rather than confirmatory, and prospective randomized controlled studies are needed to further clarify the comparative efficacy of these techniques.

## 5. Conclusions

In this retrospective cohort, TAP, QL, and C-TPV blocks were associated with low perioperative opioid requirements and comparable safety profiles in dogs undergoing laparoscopic ovariectomy. C-TPV was associated with a lower proportion of dogs requiring intraoperative rescue opioids, whereas QL showed the shortest execution time.

Postoperative differences were minimal and of uncertain clinical relevance. These findings should be interpreted with caution due to the retrospective, non-randomized design of the study. Prospective controlled studies are warranted to confirm these results.

## Figures and Tables

**Table 1:** Baseline and perioperative variables in dogs undergoing laparoscopic ovariectomy receiving TAP, QL, or C-TPV blocks. Data are reported as median and interquartile range (IQR). *p* values refer to overall between-group comparisons. Pairwise comparisons are shown for variables with significant overall differences. Significant differences were observed for age, dexmedetomidine dosage, and block execution time, whereas body weight and methadone dosage did not differ significantly among groups.

Variable	TAP	QL	C-TPV	*p* Value
Age (years)	2.0 (1.5–3.0)	1.75 (1.0–3.0)	3.0 (2.0–6.0)	0.01
Body weight (kg)	19.0 (13.0–30.0)	19.0 (8.9–33.0)	20.0 (12.0–30.0)	0.91
Methadone (mg kg^−1^)	0.2 (0.2–0.2)	0.2 (0.2–0.2)	0.2 (0.2–0.2)	0.15
Dexmedetomidine (µg kg^−1^)	4 (3–4)	4 (4–4)	3 (3–4)	<0.01 Pairwise comparisons: QL vs. TAP: 0.09 QL vs. C-TPV: 0.09 C-TPV vs. TAP: <0.01
Block execution time (s)	78 (46–82)	41 (39–47)	57 (52–62)	<0.01 Pairwise comparisons: QL vs. TAP: <0.01 QL vs. C-TPV: 0.03 C-TPV vs. TAP: <0.01

**Table 2:** Incidence of block-related complications and hypotension in dogs undergoing laparoscopic ovariectomy in the TAP, QL, and C-TPV groups. Data are reported as number of cases (percentage). Hypotension was evaluated both within 20 min after block performance and during the intraoperative period. No statistically significant differences were observed among the groups.

Variable	TAP	QL	C-TPV	*p* Value
Complications	2 (6%)	0 (0%)	1 (3%)	0.39
Hypotension within 20 min after block performance	2 (6%)	0 (0%)	0 (0%)	0.06
Intraoperative hypotension	6 (18%)	4 (11%)	5 (17%)	0.68

**Table 3:** Proportion of dogs requiring rescue analgesia during the intraoperative and postoperative periods in dogs undergoing laparoscopic ovariectomy receiving TAP, QL, or C-TPV blocks. Data are reported as number of dogs requiring at least one rescue administration [*n* (%)] within each group. Pairwise comparisons between groups are reported as indicated.

Variable	TAP	QL	C-TPV	*p* Value
Dogs requiring at least one intraoperative fentanyl rescue, *n* (%)	27/33 (81.8%)	13/36 (36.1%)	0/29 (0%)	<0.01 Pairwise comparisons: QL vs. TAP < 0.01 QL vs. C-TPV < 0.01 C-TPV vs. TAP < 0.01
Dogs requiring at least one postoperative methadone rescue, *n* (%)	7/33 (21.2%)	4/36 (11.1%)	0/29 (0%)	0.03 Pairwise comparisons: QL vs. TAP 0.33 QL vs. C-TPV 0.18 C-TPV vs. TAP 0.04

## Data Availability

The original contributions presented in this study are included in the article. Further inquiries can be directed to the corresponding author.
